# Payments by Patients

**Published:** 1920-08-28

**Authors:** 


### HOSPITAL QUESTIONS IN AUSTRALIA.

### Payments by Patients.

(By a Special Correspondent.)

The question of having paying wards in connection with hospitals erected solely for the treatment of the sick poor is one about which much may be said for and against The experiment was tried at one of the largest general hospitals in Australia about thirty-five years ago, when two classes of paying patients, in addition to ordinary non-payers, were catered for. Some ten private rooms were set apart for what were designated Class "A" patients, while two wards containing ten beds each were devoted to male and female patients respectively in the Class "B" division. The fees charged the former were £5 5s. and the latter £3 3s. a week. From these amounts one guinea a week was deducted and pooled and distributed every quarter equally amongst all members of both the honorary and resident medical staffs of the hospital, to which amount was added all operation fees according to a sliding scale—*e.g.*, an operation, say, for hæmorrhoids, would be £10 10s. for a Class "A" patient, and half that amount for a Class "B" patient.

No questions were asked the patrons of these wards as to their financial circumstances; consequently many well-to-do people were treated therein. But in extenuation of this it should be remembered that at that time the number of private hospitals in the Metropolis referred to could be counted on the fingers of one hand. A Royal Commission, appointed by the Government to inquire into the working of hospitals and charities generally reported unfavourably on having paying wards as an adjunct to public hospitals, and consequently the system was abolished. But the financial crash that occurred in Australia in 1893 necessitated the adoption of such drastic economy that either the number of beds had to be seriously reduced or means devised to increase revenue substantially.

The result was that "patients' contributions," hitherto a negligible quantity, became from that time forward a substantial and ever-increasing source of income, and now, in the case of the hospital alluded to, constitutes one-fourth of the annual maintenance receipts. Thus, in the year ended June 30, 1919, out of £21,000 received, £5,300 was "from or on account of patients." And this is not a record amount.

This result was due to unremitting systematic inquiry and solicitation. Thus every patient seeking indoor or outdoor treatment is either personally or by proxy interrogated as to his or her means. In the case of in-patients, the following are some of the questions asked in addition to the usual formal ones as to age, address, occupation, etc.—viz.: Average weekly earnings for past six months? Are you a member of a Lodge or in receipt of any pension? Amount and value of property (if any), or money in a bank? Rent of dwelling? Doctor's fees paid or owing in previous six months? Family earnings? Name of reference who can confirm declaration that you are unable to pay medical fees. These particulars, together with amount of contribution promised, are then signed as a solemn declaration by or on behalf of the patient. When the patient is discharged, if unable to pay the amount due as promised, the question is asked, "When will you pay?" and the answer noted. In default of payment at expiration of stated period, the patient is reminded by letter, and if no reply is vouchsafed, then, if amount and accessibility of defaulter warrant it, the services of the hospital collector are enlisted.

The amounts accepted from in-patients vary from "a donation of 5s. after treatment" to weekly payments ranging from 2s. 6d. to 42s. The Government rightly enough lays down a rule that the amount demanded shall not exceed the average cost of occupied beds for the previous financial year. Those who are able to pay more than this are judged suitable cases for intermediary or private hospitals. In regard to out-patients, the fees range from 1s. to 5s. a week, according to circumstances. For those suffering from venereal diseases who attend the night clinic, a minimum fee of 5s. a week is asked (but not always obtained), for these are men generally in work, who have not to sacrifice any pay by hospital attendance during working hours.

A good many vigorous protests have from time to time been made by certain members of the medical profession against the system of patients' contributions, and some have gone so far as to assert that no fee whatever should be paid by any indoor or outdoor hospital patient. But this is an extreme view to take, and one that is not likely to commend itself to an unbiased mind. The writer has championed the system for over a quarter of a century, believing it to be the most legitimate source of hospital revenue, and one that has a good moral effect, inasmuch as the donor of a contribution towards his treatment or maintenance is not pauperised, and can retain his self-respect. For a long time the Victorian Government did nothing to encourage the development or general adoption of the system, but of late years has veered round, and now insists upon all patients being required to give as much as they can afford. As regards the objections raised by some doctors, it may be pointed out that the high fees charged in cities like Melbourne and Sydney for medical treatment and surgical operations drive scores of middle-class people into our public hospitals; while, on the other hand, it is a well-known fact that some doctors are very keen to get rid of troublesome lodge patients by running them into a charitable institution.

